# Structure of allergens and structure based epitope predictions^☆^

**DOI:** 10.1016/j.ymeth.2013.07.024

**Published:** 2014-03-01

**Authors:** Fabio Dall’Antonia, Tea Pavkov-Keller, Klaus Zangger, Walter Keller

**Affiliations:** a European Molecular Biology Laboratory, Hamburg Outstation, Hamburg, Germany; b ACIB (Austrian Centre of Industrial Biotechnology), Petersgasse 14, 8010 Graz, Austria; c Institute of Molecular Biosciences, University of Graz, Austria; d Institute of Chemistry, University of Graz, 8010 Graz, Austria

**Keywords:** Allergen structure, Protein family, X-ray, NMR, IgE epitope, Structure based epitope prediction

## Abstract

The structure determination of major allergens is a prerequisite for analyzing surface exposed areas of the allergen and for mapping conformational epitopes. These may be determined by experimental methods including crystallographic and NMR-based approaches or predicted by computational methods. In this review we summarize the existing structural information on allergens and their classification in protein fold families. The currently available allergen-antibody complexes are described and the experimentally obtained epitopes compared. Furthermore we discuss established methods for linear and conformational epitope mapping, putting special emphasis on a recently developed approach, which uses the structural similarity of proteins in combination with the experimental cross-reactivity data for epitope prediction.

## 1 Introduction

The three-dimensional structure of clinically relevant allergens is of central importance: (i) It allows the visualization and analysis of surface exposed residues and in combination with experimental or computational methods the actual or putative B-cell epitopes can be elucidated. (ii) Structure can yield information about bound ligands (proteins and/or small molecules), which may modulate the protein’s allergenicity. (iii) The allergen structure forms the basis for the rational design of hypoallergenic derivatives, which may be generated through various methods (point mutations, truncations, mosaic proteins, fusion with carrier proteins, etc.).

Most allergens are relatively small, stable and well-structured proteins. Therefore, they are perfectly suited for structural studies by both X-ray crystallography and NMR spectroscopy.

There are also a few examples of obviously unstructured proteins that act as allergens. In particular caseins, which based on NMR and circular dichroism (CD) evidence, are intrinsically unstructured. However, these unstructured allergens might get structured upon interactions with other proteins and/or ligands. In recent years the number of allergen structures deposited in the protein data bank increased exponentially. With the growing number and variety of structures it became clear that there was no “allergen specific fold” emerging. Rather allergens comprised a wide variety of secondary structure compositions and tertiary folds. However, as the structures of many major allergens from representative allergen sources became available it is also becoming clear that most major allergens will be grouped into a limited number of fold and functional families. Here we give an overview of all known allergen structures and their affiliation with known fold families, defined in the PFAM database.

In addition we summarize the techniques used for experimental and computational characterization of conformational epitopes. This part is complemented with an analysis and discussion of the actual knowledge on conformational epitopes gained from the structure of allergen-Fab complexes.

## 2 Structure determination of allergens

### 2.1 Crystallographic methods

Type I allergens are proteins of various physicochemical properties and very diverse primary structures and three-dimensional folds. The only common property which has emerged from the characterization of a wide variety of inhalant and food allergens is that the majority exhibits a high solubility in aqueous media. Therefore, allergens may be treated like any soluble protein when it comes to crystallization and crystal optimization, crystallographic data collection, structure solution and refinement. Well established methods exist for all of these steps on the way to the final 3D structure. Here we shall focus on techniques, which are somewhat specific for the structure determination of allergens or have been applied successfully to important allergens that resisted structure determination in their native form.

Obtaining well diffracting crystals is still the bottleneck of structure determination by crystallography. One of the most important criteria for crystallizability of a protein is the correct fold, its monodispersity and its stability. The method of choice for determining the fold and thermal stability is CD-spectroscopy and its applications to proteins and allergens has been described [1–3]. To increase the solubility of target protein various optimization procedures may be applied (e.g. the sparse matrix approach [4], additive and detergent screens) and in combination with the Thermofluor method [5] they allow for the parallel screening of vastly different conditions. In cases where the allergen is highly soluble and well ordered, but still refuses to crystallize, flexible ends or linkers between ordered domains can be present, as for the case of Phl p 5. Here it will be necessary to determine the flexible regions by experimental (e.g., limited digests) or computational methods [6,7]. Alternatively, point-mutants with changes of surface exposed residues may be necessary to promote beneficial crystal packing interactions [8]. After engineering the protein to remove the flexible parts either the truncated full-length allergen or its folded domains are submitted to crystallization.

**Fig. 1:**
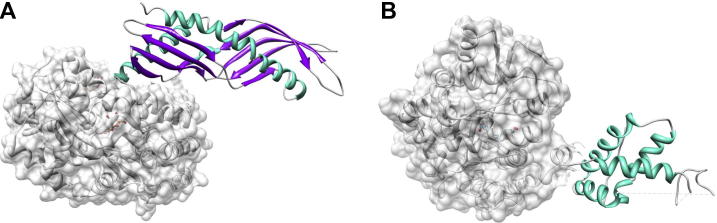
MBP-fusions act as crystallization chaperons. (A) Der p 7 (PDB: 3h4z) and (B) Ara h 2 (3OB4) are shown as maltose-binding protein (MBP)-fusion proteins. Allergen structures are shown in ribbon representation and are colored according their secondary structure composition (α-helices in cyan, β-sheets in magenta). MBP is additionally shown as surface representation (gray). Dashed lines indicate the disordered regions that are missing in the crystal structures.

A quite different approach which has been applied to the structure determination of allergens is the use of a fusion chaperon, where the smaller allergen is fused to a larger fusion partner, which has been shown (or even optimized) to promote crystallization [6]. This approach was successful for the structure determination of two allergens: Der p 7 and Ara h 2 (Fig. 1) [9]-[10]. Finally, the use of a specific binding partner (e.g., Fab or Fv) for complex formation can also enhance the crystallizability of the allergen – the Fab acts as a non-covalent crystallization chaperon. In addition the complex structure yields the exact information about the binding site (discussed in detail in Section 4.2).

### 2.2 NMR methods

#### 2.2.1 Preparation of protein

The very first NMR studies on allergen structures were carried out using proteins isolated from natural sources. In particular, the ragweed allergen Amb t 5 (previously called Ra5 and Amb t V) structure was determined using homonuclear experiments on isolated protein at natural isotopic abundance [11–13]. Because of the large protein amounts needed and to enable isotopic enrichment, recombinant proteins were used for most other NMR studies of allergens. Therefore, the cDNA of the allergen or a synthetic DNA corresponding to the desired protein is cloned typically into overexpression vectors of *Escherichia coli* cells. While rich media (e.g., LB broth) can be used for protein expression at natural abundance, minimal medium containing ^15^NH_4_Cl as nitrogen and ^13^C–glucose as carbon source are typically used.

#### 2.2.2 NMR assignment and structure determination

##### 2.2.2.1 At natural abundance

Due to the limited spectral dispersion of ^1^H NMR spectra, structural protein NMR studies on allergens at natural isotopic abundance are limited in size to <∼15 kDa. Chemical shift assignment of ^1^H nuclei (protons) is achieved by first identifying spin systems of individual amino acids in a 2D TOCSY spectrum and subsequently establishing sequential connections via short through-space proton-proton distances (NOEs or Nuclear Overhauser Enhancements) [14]. This approach has been employed to obtain the structures of Amb t V (5 kDa) [11] and Phl p 2 (11 kDa) [15]. Due to the low spectral resolution of ^1^H and ambiguities in using NOEs for sequential assignment nowadays almost all proteins used for NMR structural studies are labeled with stable isotopes to circumvent these difficulties.

##### 2.2.2.2 Using isotopically enriched protein

The use of proteins enriched with ^15^N and ^13^C allows the use of these additional NMR active isotopes in the assignment and structure determination approach. Both nuclei offer a much better spectral resolution and relaxation behavior (narrower line-width) than protons and the direct connectivities by chemical bonds allows the signal assignment to proceed via through-bond (scalar couplings) rather than sometimes ambiguous through-space proton-proton distances (NOEs). ^1^H, ^15^N and ^13^C resonances can be assigned using standard 3D triple-resonance experiments, which allow the sequential walk along the backbone by connecting the chemical shifts of backbone amide N and H, Cα, Cβ and C′ of a certain amino acid (i) with the corresponding frequencies of its two sequential neighbours (*i* − 1 and *i* + 1) [16]. Side-chain proton and carbon assignment is then achieved using experiments that correlate them to the previously assigned backbone nuclei using e.g., HCCONH, CCONH and HCCH TOCSY spectra. Once almost complete ^1^H, ^13^C and ^15^N assignment is accomplished structural restraints need to be acquired. As for the vast majority of proteins, also for allergens these have been mainly NOEs, but also dihedral angle restraints obtained from three-bond coupling constants and for some more recent studies orientational restraints from dipolar couplings in weakly aligned media [17]. For an accurate 3D structure determination a large number of restraints (>1000 for a protein of 10 kDa or above) is needed. Due to increasing line-width and number of signals at higher molecular weights a complete atomic-resolution 3D structure determination by NMR spectroscopy faces a size-limit which is currently around 40 kDa. For large proteins (>30 kDa) the use of TROSY-type (Transverse Relaxation Optimized Spectroscopy) [18] experiments is preferred which results in narrower lines and higher intensities.

#### 2.2.3 Information about protein dynamics

One of the advantages of NMR spectroscopy is that in addition to the structure also the dynamical behaviour of a protein can be investigated. In particular, backbone amide ^15^N T_1_ and T_2_ relaxation times as well as heteronuclear {^1^H}^15^N NOEs are used to obtain information about dynamics on the nanosecond to millisecond time-scale [19]. Additionally, solvent PREs (paramagnetic relaxation enhancements) provide details about the solvent accessibility of individual nuclei and can be used to identify flexible, solvent accessible loops in proteins [20].

#### 2.2.4 Protein–protein and protein–ligand interactions

A series of NMR experiments are available to provide information about the binding site and structure of protein–protein and protein–ligand interactions [21,22]. If a protein complex is below the size limit of NMR and the dissociation constant small enough (typically *K_d_* < ∼10^−4^ M) its three-dimensional structure can be obtained following regular NMR structure determination protocols as described above. Especially for weak interactions chemical shift mapping can be used to determine the dissociation constant and binding site on the allergen. Thereby, 2D heteronuclear correlation spectra (typically ^15^N, ^1^H-HSQCs) of the isotopically labelled free allergen are acquired and with different amounts of potential binding partner. The equilibrium between free and bound allergen leads to chemical shifts which are averaged depending on the ratio of free/bound protein. Therefore, titrating the binding partner to the allergen leads to “moving” signals in the spectra. By this method a large number of potential ligands can be screened in relatively short time. The relative shifts as a function of allergen and binding partner concentration allow for a determination of the binding constant and, if the allergen signals are assigned, provide the binding interface. On the other hand isotopically labelled ligands can be identified if the allergen is unlabelled. This approach has been used successfully to “fish” for potential ligands of Der f 2 in an isotopically labelled *E. Coli* lysate [23]. Antibody-binding epitopes on allergens can also be mapped by comparing hydrogen/deuterium exchange rates of free and antibody bound allergens [24]. Therefore, typically a 2D ^1^H, ^15^N-HSQC is acquired of the allergen in H_2_O and then the buffer changed to D_2_O. Signal reductions are indicative of chemical exchange between NMR-active ^1^H and silent deuterium. An antibody bound to an allergen leads to reduced exchange rates by steric protection of the epitope from the aqueous environment. Rather qualitative information on the binding site can also be obtained by saturation transfer experiments [25]. Thereby, a signal of the antibody is irradiated with radio-frequency and the resulting saturation is then transferred to the bound allergen, where it can be detected through a reduction in signal intensity by standard 2D NMR experiments.

#### 2.2.5 Dynamics of allergens

**Fig. 2:**
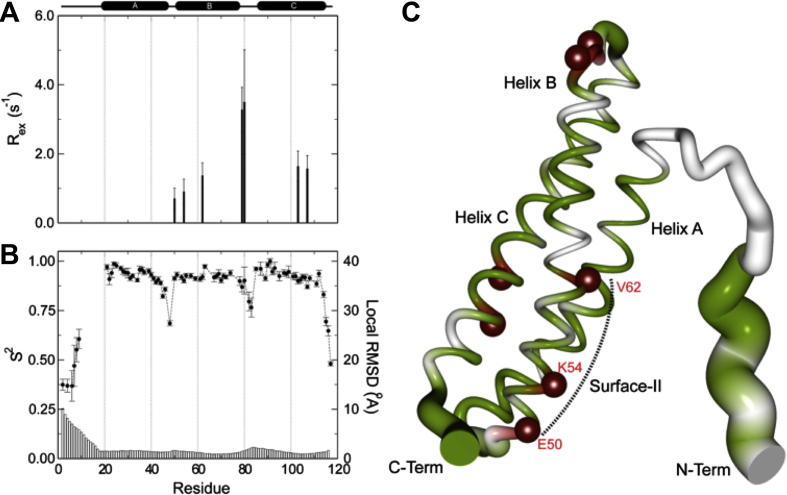
Information on chemical exchange (*R*_ex_) on the millisecond-microsecond time scale (A) and order parameter S^2^ (B) as derived from ^15^N relaxation measurements are shown as a function of residue number of Blo t 5. In (C) these parameters are mapped onto the sausage structure of Blo t 5 where the thickness is related to 1 − S^2^ and residues with *R*_ex_ contributions are represented as red spheres. Reproduced with permission from [29].

In addition to the structure also the dynamical behavior of proteins often provides clues towards their functions. The flexibility of allergens has been repeatedly suggested to be important for their allergenicity. Compared to other proteins, allergens are remarkably well-structured. However, for many allergens stretches of increased flexibility and even intrinsically unstructured regions have been identified. Unstructured regions are typically missing in X-ray structures and are characterized by poorly defined NMR structure bundles and differences in their relaxation behavior compared to well-structured parts. Relatively large unstructured regions were found for example in the mugwort pollen allergen Art v 1 [26], the tropical mite allergen Blo t 5 [27] and the olive tree pollen allergen Ole e 6 [28]. In contrast to IgG, IgE binds mainly to structured proteins. Consequently IgE epitopes have only been found in structured regions of allergens. However, the identified allergen epitopes often include somewhat flexible regions of the proteins, for example loops. Based on a model-free analysis of ^15^N relaxation data Naik et al. [29] found conformational exchange in the microsecond to millisecond timescale on the epitope surface of Blo t 5 (Fig. 2) and predicted a potential role of such motions as a general requirement for allergenicity. On the other hand this allergen is very stable on the nanosecond-picosecond time range based on higher generalized order parameters S^2^ in the antibody interaction site. The mobility of allergens is often significantly reduced by the formation of disulfide bonds, like in Ole e 6 [28], Ara h 6 [30] or Amb t 5 [11] or by the binding of e.g., calcium ions as for Bet v 4 [31].

## 3 Structural families of allergens

Allergens are a diverse group of proteins with different structures and biological functions [32–39]. To date more than a hundred entries of non-redundant allergen structures (i.e., dismissing isoforms and point mutants) have been deposited in the protein data bank (PDB). Therefore a description of each allergen structure is impractical. Here we focus on a description of the major allergen families and domains as identified in the Pfam database [40] and we add a rather complete listing of all allergen structures currently available in the PDB in Tables 1 and 2.

### 3.1 Pathogenesis-related (PR-10) protein/Bet v 1 family

**Fig. 3:**
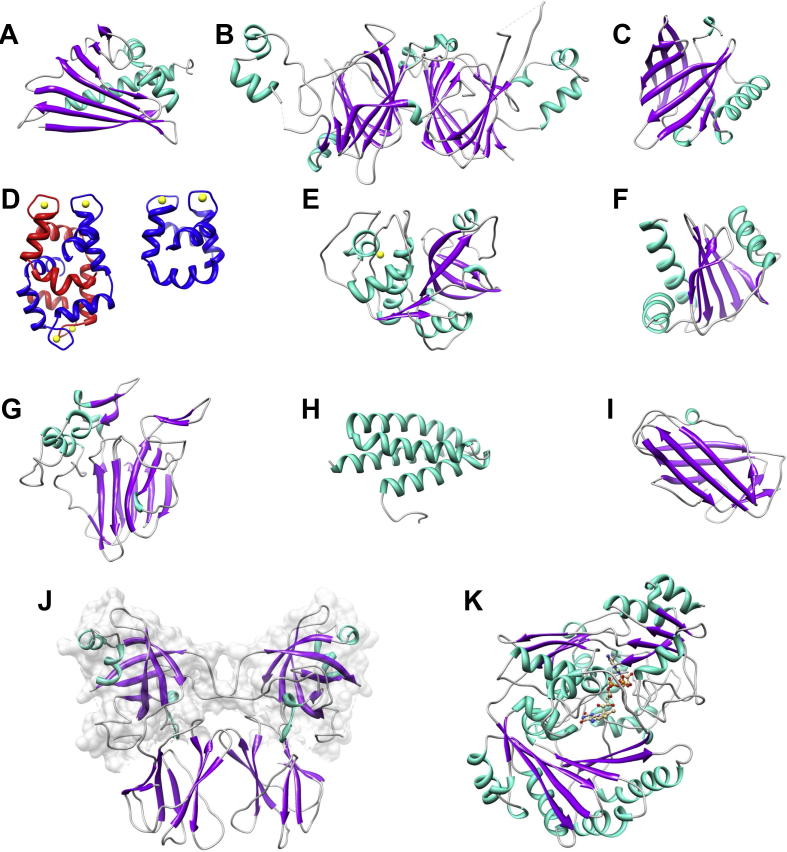
Representative structures of important allergen families. Allergen structures are presented according to their secondary structure composition (α-helices in cyan, β-sheets in magenta). A: Bet v 1 (PDB: 1BV1), B:Ara h 1 (3S7E), C: Bos d 2 (1BJ7), D: Phl p 7 (1K9U, 2LVK), E: Der p 1 (3F5 V), F: Ara h 5 (4ESP), G: Pru av 1 (2AHN), H: Phl p 6 (1NLX), I: Der p 2 (1KTJ), J: Phlp1 (1N10) and Phl p 4 (3TSH). Ca^2+^ atoms are shown in yellow and FAD cofactor (K) in ball-and-stick representation. (D) Comparison of Phl p 7 dimer (as observed in the X-ray structure) and monomer (as present in the NMR structure). (J) Allergen Phl p 1 belonging to the expansin family consists of 2 domains and forms a dimer. The N-terminal domains are distinguished as grey surface representation.

Members of this family belong to pathogenesis-related protein family PR-10. They are expressed at a high level in pollen, seeds and fruit tissue. Bet v 1 is the prevalent pollen allergen from birch and cross-reactive allergens are found in pollen of related trees (alder, hazel, and chestnut) as well as in fruits (e.g., apple, pear, and stone fruits) and vegetables (e.g., celery, carrot) [38]. The Bet v 1 family is one of the structurally best-characterized allergen families. Since the determination of the first Bet v 1 structure (Fig. 3A) by crystallography and NMR (PDB: 1BV1, PDB: 1BTV; [41]) numerous structures of Bet v1 isoforms and mutants as well as Bet v 1 related proteins from various fruits and vegetables have been determined (Table 1).

### 3.2 Protease inhibitor/seed storage/lipid transfer proteins (LTP) family

Members of this family belong to the prolamin superfamily, whose main characteristic is the presence of a conserved pattern of cysteine residues CX*_n_*CX*_n_*CCX*_n_*CXCX*_n_*CX*_n_*C that form three or four intra-molecular disulfide bonds and render these proteins very stable [36,42–45]. In the PF00234 family the disulphide bonds are formed between Cys_1_–Cys_5_, Cys_2_–Cys_3_, Cys_4_–Cys_7_, Cys_6_–Cys_8_. These disulphide bonds stabilize the central core formed by a four α-helical bundle with an internal cavity where lipophilic molecules can bind (Fig. 1B, structure of Ara h 2). A very well conserved overall fold is also present in the available structures of allergens belonging to this family. However superposition of individual molecules show significant differences between the structures concerning the loop regions as well as the placement of the helices [46]. These proteins can act as food, environmental and occupational allergens [45].

### 3.3 Cupin superfamily

The conserved β-barrel domain of the functionally very diverse ‘cupin’ superfamily represents this family of proteins [47]. Among others, it contains 11S and 7S plant seed storage globulins [48]. The 11S globulins, also called legumins, are hexameric proteins with each subunit synthetized as a single chain of ca. 60 kDa [49]. After posttranslational processing a 30–40 kDa acidic chain is linked by a disulphide bond to a 20 kDa basic chain [50]. For the allergen Gly m 6, also termed glycinin, several isoforms and five major subunits have been identified [49,51]. Other allergen structures reported for 11S globulins are Ara h 3 from peanut and Pru du 6 from almond. On the other hand, mature 7S globulins or vicilins contain three subunits forming a disk shape structure with no disulfide bond present [48]. Ara h 1 (Fig. 3B) and conglycinin, allergens from peanut and soybean belong to the 7S globulins.

### 3.4 Lipocalin/cytosolic fatty-acid binding protein family

Proteins of the lipocalin family transport small, mainly hydrophobic molecules such as lipids and steroid hormones [52,53]. This family shows a low sequence homology with conserved tertiary structure architecture (Fig. 3C): an eight-stranded antiparallel β-barrel with a short α-helical N-terminus [52–54]. The major ligand-binding site is formed by the hydrophobic cavity of the β-barrel (calyx). The lipocalin structures of mammalian respiratory allergens (mouse, cow, dog, horse and rat) have been reported. Recently the structures of arthropod lipocalins were determined, of Bla g 4 and Per a 4, which are male pheromone transport lipocalins from cockroach [55], and of Arg r 1, a histamine-binding lipocalin from pigeon ticks.

### 3.5 EF hand family

The EF hand motif is a calcium binding sequence that was first observed in the structure of parvalbumin [56] – it consists of a 12 amino acid loop region with a conserved signature and is flanked by two amphipathic helices. EF hand motifs appear to occur mostly in pairs, which may be explained by the burying of hydrophobic patches that occurs through EF-hand pairing.

A wide range of calcium-binding proteins harbor a variable count of EF hand motifs [57]: Parvalbumin are 12 kDa proteins that contain 3 EF hand motifs, the first of which is degenerate and not able to bind calcium ions [58,59]. Parvalbumins from fish are major food allergens [60]. The 2-EF hand family of allergens (also called polcalcins) consists of small 8–9 kDa proteins with two calcium binding motifs. The first structure solved of this group was that of Phl p 7 that exhibited an intertwined domain-swapped dimer, in which the N-terminal EF-hand of one monomer pairs with the C-terminal EF-hand of the second monomer (Fig. 3D) [61]. The same 3D arrangement has been found in the crystal structure of the pollen allergen Che a 3 [62]. Interestingly the solution structures of Bet v 4 [31] and Phl p 7 [63] turned out to be monomers (Fig. 3D) with intramolecular EF-hand pairing.

### 3.6 Papain-like cysteine protease

**Fig. 4:**
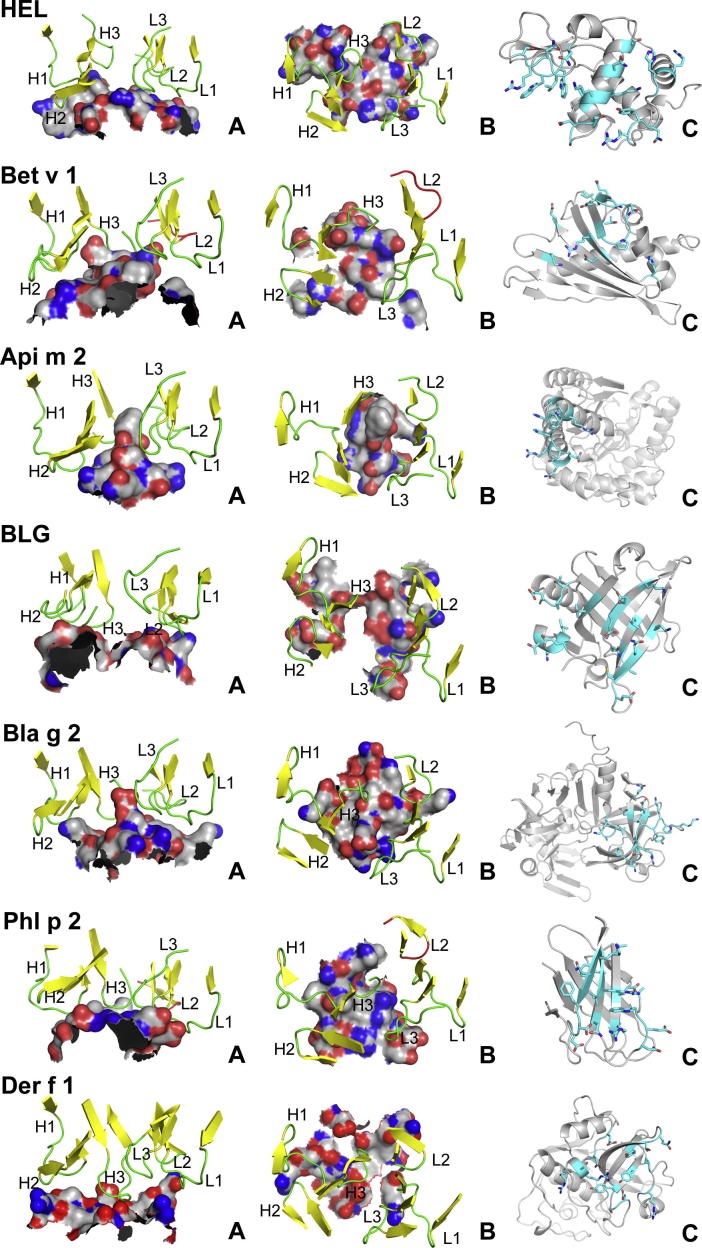
Structural aspects of allergen epitopes and Ab interfaces in complexes. PDB codes are HEL (hen egg-white lysozyme): 1NDG, Bet v 1: 1FSK, Api m 2 (bee venom hyaluronidase): 2J88, BLG (β-lactoglobulin): 2R56, Bla g 2: 2NR6, Phl p 2: 2VXQ, Der f 1: 3RVV. Epitopes in side view (panels A) and top view (B) are represented as surfaces with atom-type colors (carbon grey, nitrogen blue and oxygen red) and the interacting CDRs are shown as secondary structure cartoons with sheets in yellow and loops in green, except when not binding (red). Panels C show the entire allergens as cartoons without Ab in the same top-view orientation. Epitope residues are highlighted in cyan, and with side chain stick representation, nitrogen and oxygen colors as before. Model sizes are not on the same scale. Convexity ratios in top-down order are 0.35, 0.48, 0.69, 0.37, 0.42, 0.43 and 0.39.

The cysteine proteases are divided into several clans, each containing a number of families as reported by MEROPS, the peptidase database (http://merops.sanger.ac.uk/index.shtml) [64]. Papain-like cysteine proteases belong to the clan CA, family C1. Most members of family C1 are synthesized as inactive pro-enzymes with N-terminal pro-peptide regions with different length [65]. The pro-peptide is required for the proper folding of the enzyme and the inactivation of the peptidase domain. The catalytic residues of family C1 have been identified as Cys and His. The mature allergens Act d 1, Car p 1, Der p 1 (Fig. 3E) and Der f 1 (Fig. 4G) from kiwi fruit, papaya and house dust mites show conserved structures with two interacting domains. A catalytic site is situated in the cleft created by the two domains. One domain consists mostly of α-helices and the other of 5 β-strands and 2 α-helices. All four allergen structures have 3 disulfide bonds, from which 2 are conserved in all of them. The third disulfide bond is conserved in Act d 1 and Car p 1, and Der p 1 and Der f 1. The pro-region of Der p 1 (PDB: 1XKG) consists of 4 α –helices where the two N-terminal helices form a distinct domain. This entire pro-region is covering the binding cleft on the surface of the protein [66].

### 3.7 Profilin family

Plant profilins are highly conserved 12–15 kDa proteins [67]. The main fold consists of a central seven-stranded anti parallel β-sheet and two α-helices on each side (Fig. 3F, structure of Ara h 5). Only plant profilins are recognized as allergens.

### 3.8 Thaumatin-like protein

Thaumatin-like proteins (TLPs) are a big family of proteins sharing sequence similarity with thaumatin, the intensely sweet-tasting protein isolated from the seeds of the plant *Thaumatococcus daniellii* [68]. TLPs can be referred to as pathogenesis-related proteins (PR-5 family) [69]. Many fruits and pollen TLPs have been identified as allergens [69–74]. TLPs share a hight sequence similarity and most of them have 16 conserved cysteines, which form 8 disulfide bridges contributing to stability and proper fold of the proteins. TLPs consist of three typical domains: (I) an N-terminal core domain built by two antiparallel β-sheets interconnected by loops and organized in a flattened β -sandwich, (II) a disulfide-rich domain consisting of short α-helices connected by loops formed due to cysteine, (III) a domain comprising of two β -strands and two loop regions [74,75]. Together, domains I and II form a large cleft region on the protein surface (Fig. 3G, structure of Pru av 2).

### 3.9 Group 1/2/3 grass pollen allergen; Expansin family

Expansins are ubiquitous plant cell wall proteins and have the proposed functions of cell wall loosening, expansion and other developmental modifications [76]. Genes of the subgroup β-expansins are expressed exclusively in the pollen and are suggested to function in the directed pollen tube groth. Group 1 pollen allergens are a subgroup of the expansins and consist of two domains: domain-1 exhibits a double-psi beta-barrel (DPBB) fold [77] and domain-2 exhibits an Ig-like fold [78]. Phl p 1 forms dimers in the crystal and the glycosylated and unstructured N-terminal tail is involved in dimer formation (PDB: 1N10, Fig. 3J). Another structure from the expansin family has been reported for Zea m 1, also called EXPB1 (PDB: 2HCZ, [79]). The protein exhibits 58% identity on the amino acid sequence level, but IgE cross-reactivity with Phl p 1 has not been proven.

Proteins from the group 2/3 pollen allergens share high sequence and structure similarity with the C-terminal domain of expansins, but lack the N-terminal domain. Structure representatives of both groups have been determined by crystallography as well as by NMR [15,80]. In addition a Phl p 2-Fab complex has been determined (PDB: 2VXQ, [81]), defining a specific IgE epitope (see chapter 4.2).

### 3.10 Expansin, N-terminal domain

The members of this family are quite diverse and this domain is found at the N terminus of some pollen allergen, like Phl p 1 (Fig. 3J) and Zea m 1. The domain fold is dominated by a six-stranded β-barrel flanked by short loops and α–helices, also termed double-psi β-barrel (DPBB) [77,79]. N-terminal domains of Phl p 1 and Zea m 1 have 8 conserved cysteines involved in three disulfide bonds.

### 3.11 Group 5/6 grass pollen allergen

Group 5 grass pollen allergens (GPA) are major allergens eliciting specific IgE reactivity in sera of 65 – 90% of GPA allergic patients. The protein consists of two well folded domains separated by a 20 amino acid linker and an unstructured N-terminal tail, as determined by secondary structure prediction. Crystals have been obtained of the recombinant Phl p 5b protein (current name according to IUIS nomenclature: Phl p 5.0201), which had a molecular mass of 29 kDa. However, the crystals contained only the C-terminal 13 kDa fragment [82]. The structure was solved yielding a disulfide bridged dimer of the C-terminal domain (PDB: 1L3P, [83]). The protein is mainly α-helical and each monomer contains a 4-helix bundle. A similar structure was found for Phl p 6 (PDB: 1NLX), which has a molecular mass of 11.8 kDa and contains only one domain with a four-helix bundle (Fig. 3H). According to sequence alignments Phl p 6 is closer related to the N-terminal domain of group 5 allergens.

### 3.12 Group 5/21 mite allergen

Group 5 allergens from dust mites *Blomia tropicalis* and *Dermatophagoides pteronyssinus,* Blo t 5 and Der p 5 belong to a group of α-helical proteins (Fig. 2C). The structure is comprised of three helices arranged in an antiparallel fashion [27,29,84]. However, two reported NMR-structures of Blo t 5 (PDB: 2JMH and 2JRK) show different topologies and different helix orientations. The fold topology of the Der p 5 reported for the X-ray structure (PDB: 3MQ1) is like the one observed in the 2JMH structure suggesting that this is the correct fold for the group 5 proteins [84].

### 3.13 CRISP; PR-1; antigen 5 (Ag5)

Antigen 5 (Ag5) allergens Sol i 3 and Ves v 5 belong to a large family of eukaryotic extracellular proteins, the CAP superfamily, where CAP is the acronym for cysteine-rich secretory proteins, antigen 5 and pathogenesis-related 1 proteins [85,86]. Their overall structure shows an α-β-α-sandwich arranged in three stacked layers: the upper layer consist of three α-helices, the middle accommodates the four-stranded anti-parallel β-sheet and the lower layer consists of two α-helices [86,87]. Most of the CAP proteins contain several cysteines, which are involved in disulfide bond formation [86]. Sol i 3 and Ves v 5 allergens are stabilized by 4 disulfide bonds.

### 3.14 Chitin recognition protein; Hevein-like domain

Allergens Hev b 6.02 (hevein) and Tri a 18 (agglutinin from wheat germ) belong to the carbohydrate-binding module family 18 (CBM18), also termed chitin binding 1 or chitin recognition proteins [88]. They comprise of a hevein-like domain that consists of 43 amino acid residues and 4 conserved disulfide bonds. In Tri a 18 four hevein-like domains are present [89]. It is suggested that this domain is involved in recognition or binding of chitin subunits [88]. Hev b 6.02 and hevein-like domain from Tri a 18 show 66% sequence similarity.

### 3.15 MD-2-related lipid-recognition (ML) domain; Group 2 mite allergen

Allergens Der p 2 (Fig. 3I) and Der f 2 share a high sequence homology [90] and posses a typical immunoglobulin fold. This fold consists of 8 β-strands and is characteristic for the immunoglobulin superfamily [91]. There are three conserved disulfide bonds present forming covalent bonds between residues 21 and 27, 73 and 78, and 8 and 119. For both allergens, NMR and crystal structures are determined (see Table 1). They differ in the reported number of strands, the position of the 2 β-sheets to one another and existence (or absence) of an internal cavity, which can accommodate a hydrophobic ligand [90,92–94].

### 3.16 Glycosyl hydrolases family 17; endo-beta-1,3-glucosidase

Familiy 17 of glycosyl hydrolases belongs to the large superfamily of TIM-barrel glycosyl hydrolase. A classification of these enzymes can be found in the CAZy database (Carbohydrate-Active enZYmes) [95]. Allergens Hev b 2 and Mus m 5 are endo-1, 3-beta-glucosidase belonging to glycosyl hydrolase family 17. Their crystal structure display the typical (α/β)_8_ TIM-barrel motif. Two catalytic glutamates situated in the center of an extended groove are proposed to act as proton donor and nucleophile residues [96].

### 3.17 Hyaluronidase

Hyaluronidases belong to the family 56 of glycosyl hydrolases [95] and degrade a large linear polymer, hyaluronic acid [97]. The structures of two hyaluronidases from honeybee and common wasp venom, Api m 2 (Fig. 4) and Ves v 2, reveal a central (α/β)_7_ core with a large cavity near the active site that is involved in substrate binding [98,99]. Two disulfide-bonds are conserved in both structures. The N-glycosylation has been suggested to be important for the cross-reactivity of these two allergens [100] and some of the putative N-glycosylation sites could be experimentally determined [99,101,102].

### 3.18 Cyclophilin type peptidyl-prolyl cis–trans isomerase / CLD

Cyclophilins belong to the group of proteins that have peptidyl-prolyl *cis*–*trans* isomerase activity [103]. They share a domain of about 109 amino acids that consist of an eight-stranded antiparallel β-barrel and two α-helices covering the top and the bottom of the barrel. Structures of two allergens from mold, Asp f 11 and Mala s 6, possess two conserved cysteine residues that could form a disulfide bond. This is the case for the structure of Asp f 11, whereas in Mala s 6 they are present in the reduced form [104,105]. Binding of the Ala-Pro peptide in the active site of Mala s 6 confirmed that the active-site residues are highly conserved among all cyclophilins [104].

### 3.19 FAD linked oxidase/BBE-like

Group 4 grass pollen allergens have been characterized as high molecular mass, glycosylated proteins. They belong to the BBE family of oxidoreductases and feature a di-covalently attached FAD cofactor. Recently two structures of this fold family have been solved: Cyn d 4 (previously called BG60) from Bermuda grass [106], isolated from natural source, and Phl p 4 [107] expressed in *P. pastoris* both showed the typical 2 domain structure consisting of FAD-binding domain and substrate binding domain (Fig. 3K). The superposition of the cross-reactive allergens showed a close fit (rmsd = 0.471 Å for 434 aligned C_α_ atoms). A significant difference was detected in the conformation of the FAD cofactor – while the FAD moiety in the Cyn d 4 structure was planar hinting at an oxidized cofactor, it exhibited a significant upward fold in the Phl p 4 structure which is indicative of a partially reduced FAD.

## 4 Epitope determination

### 4.1 Definition and classifications of epitopes

In the immunological context the term “epitope” and its synonym “antigenic determinant” are used to specify the recognition site of an antigen – that is, any part of an exogenous molecule recognized by components of the immune system such as antibodies (Abs), B-cells or T-cells. The Ab site that binds the cognate epitope is called “paratope” or “antibody combining site”. A major distinction is made between T-cell and B-cell epitopes: The former are rather short linear segments, located anywhere along the amino acid sequence of the antigen and also corresponding to buried parts of its three-dimensional fold. These epitopes are recognized after degradation, when they are presented to T-cells as peptides in complex with MHC-class molecules [108]. Contrarily, B-cell epitopes are located on the surface of the intact antigen, being initially recognized by membrane-bound immunoglobulin receptors of B-lymphocytes [109]. The recognition region formed by B-cell epitope and paratope is thus a special case of a protein–protein interface in a transient non-obligatory complex [110].

As reviewed in more detail by Benjamin et al. [111], the understanding of the B-cell epitope topology was facilitated by the early availability of crystal structures for proteins like sperm whale myoglobin, lysozyme or cytochrome C, serving as model antigens. Two terms for different topologies, namely “continuous” and “discontinuous” epitopes, were introduced by Atassi [112]. Later studies revealed that – irrespective of whether the antigenic determinant corresponds to one linear sequence segment (continuous epitope) or is assembled by several separated segments in spatial proximity (discontinuous epitope) – the native conformation of the protein is necessary for antibody recognition and binding. Therefore, peptide fragments representing continuous B-cell epitopes will usually bind Abs against the entire protein only if their dominant conformation in solution resembles the one of the native protein fold [113,114].

### 4.2 Crystal structures of allergens in complex with antibodies

The structure determination of complexes formed between monoclonal Abs (mAbs) and their protein antigens facilitates the immediate spatial characterization of epitope-paratope interfaces. This approach is in contrast to the indirect method of epitope mapping, where the results of peptide-based Ab binding and inhibition studies are projected onto the structural antigen model.

Ab-bound allergens account only for a small subset of the available structures of such complexes. Table X lists the eight representative crystal structures. Hen egg-white lysozyme (HEL) accounts for 31 structures of complexes with several mAbs, and for dust mite allergen Der p 1 in complex with mAb 4C1 two different monoclinic crystal structures were published [115]. The other six allergens were determined in unique complexes once. Two of these, β-lactoglobulin (BLG) and Phl p 2, were co-crystallized with hybrid *F*_ab_ components stemming from constant IgG domains and IgE-derived *F_v_* fragments, therefore the allergen epitopes bind to IgE-type paratopes of the respective Ab [81,116]. For the following discussion the structures PDB:1NDG [117] and PDB:3RVW [115] were chosen as HEL-Ab and Der p 1-Ab representatives, respectively.

When reviewing the original structure publications, a comparative epitope analysis is somewhat hampered by the fact that diverging criteria were applied in order to define the epitopes and their amino acid composition. For comparability a principal distinction has to be made between the structural epitope, which is the ensemble of residues in contact with the Ab at a distance below the interaction cut-off of 4 Å, and the functional epitope, which is defined by the subset of structural residues that bind the Ab directly and through specific interactions so that their mutation results in significant loss of Ab binding affinity [118]. Non-functional residues are involved in unspecific interactions like apolar Van-der-Waals contacts only. Irrespective of these two residue classes, the entire structural epitope corresponds very closely to the ensemble of residues that become partially or completely buried in the interface.

As the complex structures reveal (Table 3) the total buried areas of the interfaces – considering allergen plus Ab – are roughly in the range 1600 ± 230 Å^2^ which corresponds to sizes found for protein–protein recognition sites before [110]. Within this range, the recent structures of the Der f 1 and Der p 1 bound to the same mAb [115] exhibit the smallest interface areas. This goes along with a reduced total number of contacts and indicates a comparably loose mode of binding. Interestingly Der f 1 and Der p 1, being 81% identical in sequence, feature a very similar epitope to the cross-reactive mAb 4C1 in general, but concerning the specific hydrogen bonds, there are 2 of the homologous residues that bind only in the Der p 1 complex and another one that binds only in the Der f 1 complex.

It is a common feature of the eight compared complexes that all principal types of interaction, including ionic ones, are realized. While the contact numbers are similar on the whole, the contribution of functional residues to the epitopes differs considerably: it is particularly high for hyaluronidase and Bla g 2 with 80% and 75% of the total residue numbers, respectively, but well below 50% in the other cases. The highest overall number of hydrogen bonds is observed in the complex of cockroach allergen Bla g 2, where also several cation-π interactions between basic amino acids of the allergen and Ab tyrosine residues are found [119]. Grass pollen allergen Phl p 2 is somewhat exceptional in that water-mediated contacts have a significant contribution to the interface [81]. There are 15 directly Ab-binding epitope residues, nine of which are involved in hydrogen bonds, but six additional residues bind the paratope indirectly, bridged by water molecules. Hydrogen bonds involving backbone atoms of the allergen are found in all of the structures and in most of the epitopes two or more of the functional residues form hydrogen bonds exclusively by means of their backbone atoms. For Bet v 1 and Bla g 2 it is even the majority of epitope residues (4/7 and 7/12, respectively) that do not involve any side chain atom in these specific interactions.

**Fig. 5:**
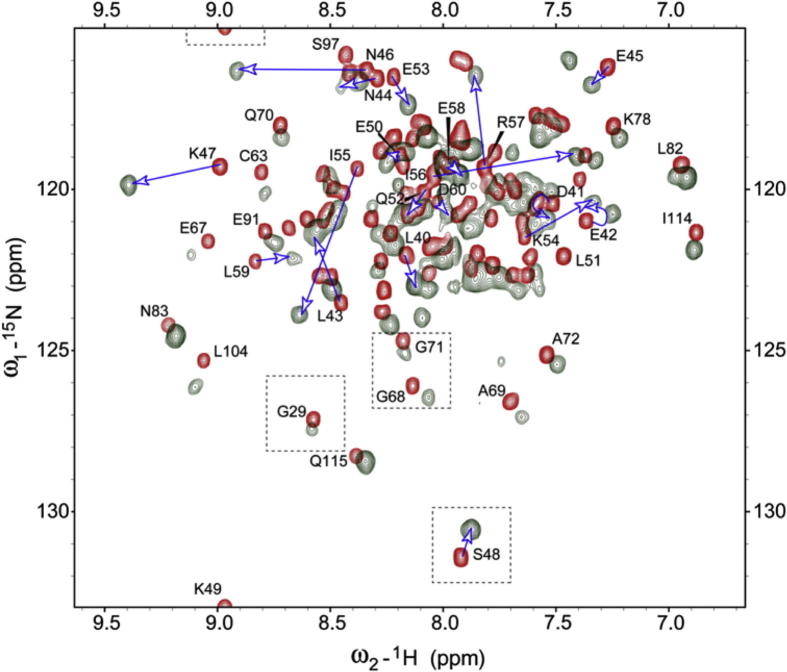
Overlay of TROSY-HSQC spectra of Blo t 5 in the absence (red) and presence (grey) of Fab. Peaks belonging to residues of the interaction region between residues 40 and 60 are indicated by arrows. Folded peaks are in dotted squares. Reproduced with permission from [29].

Except for hyaluronidase, all allergens exhibit discontinuous epitopes (Fig. 4). In the majority of cases the “classical” assembly of multiple linear segments is observed, however birch pollen allergen Bet v 1 employs only one pronounced loop of 11 continuous residues (the so-called P-loop) whereas the additional 6 interaction residues are scattered along the rest of the sequence (Figs. 4, 2C). Similarly, the epitope of Bla g 2 is dominated by one loop of 10 residues and most of the other epitope residues are isolated (Figs. 4, 5C). Bee venom hyaluronidase is the only allergen that binds the Ab by means of a truly continuous epitope, where only one sequence stretch and no additional, sequentially distant residues are involved (Figs. 4, 3C) [120]. Consequently, the total number of structural residues is smaller than for the other complexes and the interface area is comparably small. This epitope is also remarkable in that the binding loop is very protruding (Figs. 4, 3A) and three sequentially centered hydrophobic residues have a prominent position at its tip. As can be expected, this conformation is unique to the native allergen fold and peptides corresponding to the epitope segment are not able to bind the Ab. Compared to the other allergens, the loop-dominated epitopes of hyaluronidase and Bet v 1 are found to be most convex in terms of the surface shape. The IgE-binding allergens BLG and Phl p 2 have the major parts of their epitopes located on β-sheets, (Fig. 4, 4C+6C) whereas HEL is the only case with a significant contribution of α-helical amino acid residues. HEL and BLG are particularly planar, while for Phl p 2 and Bla g 2 a medium convexity can be observed. The convexity feature can be measured quantitatively by means of calculating a least-squares plane through all epitope atoms and then determining the ratio between maximum atom distances perpendicular to the plane (height of the epitope) and within the plane-projected atoms (width of the epitope). According to this formalism hyaluronidase features the most convex epitope by far with a ratio of 0.69, and HEL the most planar one with a ratio of 0.35. All values are listed in the caption of Fig. 4.

From the Ab side, molecular recognition is realized through six hypervariable regions that were originally identified by sequence variations [121]. These parts of the Ab, also called complementarity determinant regions (CDRs), were found to be loops that connect a core β-sheet framework and adopt a limited set of canonical conformations [122]. In six of the eight compared Ab-allergen complexes every CDR is involved in the interface, while Bet v 1 and Phl p 2 are bound by Abs that do not employ the second loop of the light chain (L2). As can be appreciated in Fig. 4, the spatial arrangement of the six CDRs relative to each other is very similar in all the complexes, and even the backbone conformation of the single loops is mostly alike. It can be concluded that the specificity of Ab interaction is ensured by sequence variation and flexible side chain conformation rather than major conformational backbone changes, although the CDR loop flexibility may have an additional effect.

### 4.3 NMR methods for epitope characterization

While X-ray structures between allergens and IgE antibodies provide the most accurate structural information about these interactions, several studies have reported antibody binding epitopes based on chemical shift mapping, hydrogen deuterium exchange or cross-saturation [23,26,29,123]. Chemical shift mapping arguably provides the fastest way of determining binding sites on a protein. This approach has been used successfully to determine the binding epitope of an Fab’ fragment on the tropical mite allergen Blo t 5 [29]. Due to the large size of the resulting complex TROSY-HSQC NMR spectra were acquired (Fig. 5). Differences in peak positions between free allergen and after the addition of the antibody fragment delineate the binding interface, which can be mapped onto the protein surface if the NMR signals of the free allergen are sequentially assigned. As an example for the use of hydrogen/deuterium exchange for epitope mapping, Mueller et al. found three distinct epitopes of monoclonal antibodies on the mite allergen Der p 2 [124]. For this study murine monoclonal antibodies were used and similar binding to IgE demonstrated by competitive inhibition. Until recently all studies aimed at mapping antibody binding sites on unmodified wild-type allergens used monoclonal antibodies. However, it is desirable to obtain epitope interaction sites for polyclonal antibodies. Razzera et al. [26] used chemical shift mapping between the major mugwort pollen allergen Art v 1 and patient-derived polyclonal IgE antibodies. Due to the low concentrations of the latter, the observed chemical shift differences are however very small and need to be interpreted cautiously. For the study on Art v 1 confirmation was obtained by cross-saturation between IgE and the allergen.

#### 4.3.1 Binding of other ligands

Besides antibodies, the binding of allergens to other natural ligands has been investigated and structurally characterized in order to understand mainly their physiological primary function(s). In line with the great variability of their 3D structures, allergens have been found to bind a rather diverse range of ligands. Many allergens bind hydrophobic molecules and some are part of the host’s innate immunity system. This property has been suggested to contribute to their allergenicity. Examples are the binding of short-chain fatty acids to a repeat unit of the nematode polyprotein allergen ABA-1 [125], the major mite allergen Der f 2, which binds to lipopolysaccharides as found by fishing for targets of this allergen in ^13^C-labeled *E. coli* lysates [23] and the binding of phytosteroids to the major cherry allergen Pru av 1 [126]. The birch allergen Bet v 1 which displays a large hydrophobic cleft in its structure is able to bind a broad spectrum of hydrophobic molecules including fatty acids, flavonoids and cytokinins [41]. This multitude of ligands makes it difficult to assign one unique function to Bet v 1. Another birch pollen allergen, Bet v 4, binds two calcium ions, which are required for stability of their two EF hands [31].

### 4.4 Structure-based *in silico* methods for epitope prediction

#### 4.4.1 Linear B-cell epitope prediction

##### 4.4.1.1 Concept

The linear nature of continuous B-cell epitopes has given rise to a number of prediction approaches that are based on sequence analysis methods. None of these require a structure for the actual prediction, however their development was mostly rendered possible, or at least supported, by structural knowledge. The task of predicting linear epitopes is more challenging for B-cell than for T-cell epitopes since the mode of interaction with the Ab is more complex than the sterically constrained recognition of degraded peptides in the MHC groove [127]. The original concept of B-cell epitope prediction includes the derivation of propensity scales from physicochemical features of amino acid residues and the probing of average propensity measures along the antigen sequence by means of a sliding window with certain fixed length (usually six residues). Maxima of the smoothened propensity scales are interpreted as likely epitope positions, allowing the actual prediction to be controlled by thresholds.

##### 4.4.1.2 Propensity scales

Hopp & Woods [128] employed tabulated hydrophilicity values and validated their predictions using a sequence set from 12 proteins with known epitopes, finding that the most accurate results are obtained with hexapeptide probe segments.

Westhof et al. [129] discovered a clear correlation between maxima in the sequential temperature factor profiles and the epitopes of three studied proteins when they compared the refined crystal structure parameters with the known antigenic topologies. For the tobacco mosaic virus coat protein, this correlation was in fact higher than that between antigenicity and hydrophilicity. The authors concluded that antigenicity depends on segmental mobility of the polypeptide chain. Karplus & Schulz [130] adopted this basic idea and evaluated normalized temperature factors from a set of 31 protein structures in order to derive an algorithm that predicts flexibility based on neighbor-correlated amino acid scores.

Emini et al. [131] calculated normalized segmental products of surface probabilities based on the relative frequencies of amino acid types found to be surface-exposed or buried in 28 structures [132]. The comparison of the obtained surface probability profiles of two viral proteins allowed them to align the corresponding sequences despite low conservation and to predict the epitope on the surface of the hepatitis A virus protein by mapping the known antigenic site of the poliovirus protein.

##### 4.4.1.3 Machine learning approaches

Since a study of Blythe & Flower [133] revealed that even the best single-feature-based as well as combined scales yield only marginally more accurate results than random predictions, the concept of trained classification was adopted in modern approaches. Söllner & Mayer [127] studied six classifiers based on the decision tree and k-nearest neighbor learning strategies, which were provided with up to 164-dimensional parameter sets derived from combinations of propensity scales and neighborhood matrices. Each of the classifiers was able to outperform existing methods when tested on a HIV antigen data set.

An enhancement of prediction accuracy could also be achieved with a support vector machine (SVM) [134] classifier that was provided with amino acid pair (AAP) antigenicity scales [135]. The AAP method was based on the finding that certain type pairs of sequentially neighboring amino acids within segments of length 20 are enriched in epitopes as compared to non-antigenic peptides. The best results were obtained when using multi-component vectors for the SVM, that is, by the combination of the AAP scale with several feature-based ones.

##### 4.4.1.4 Software implementations

Various implementations of linear prediction algorithms exist nowadays. An established practice is the 5-fold cross-validation using training data with epitopes from the Bcipep database [136] versus random non-epitope peptides. Some of the prediction tools combine multiple physicochemical and/or structural features: BEpitope [137] derives over 30 propensity scales from such combinations, including the detection of β-turns, and optionally adds user-defined patterns. Contrarily, ABCPred [138] ignores traditional scales and classifies amino acid patterns directly. To develop their classification model the authors employed a recurrent neural network, which was supplied with 16-residue long input sequences.

BCPred [139] combines AAP scales and the SVM classifier, similar to the work described before. The subsequence kernel technique was found to be most effective for the SVM (Kernels map input data into higher-dimensional “feature space” to enable a linear separation, as required for classification). In order to avoid overly optimistic estimations of prediction accuracy, the use of a truly non-redundant data set as obtained by homology reduction to <80% is proposed.

BepiPred [140] utilizes a Hidden-Markov-Model. Based on sequential scoring windows, a peptide matrix is constructed and evaluated to obtain epitope probability scores for each amino acid residue. These are combined with the hydrophilicity scale of Parker et al. [141] to obtain the final prediction.

Two recent tools broaden the range of linear methods: While CBtope [142] attempts to predict *dis*continuous B-cell epitopes from sequential input data, B-Pred [143] utilizes 3D homology models to identify linear epitopes.

#### 4.4.2 Structure-based prediction of (discontinuous) B-cell epitopes

##### 4.4.2.1 Principal considerations

Although linear B-cell epitope mapping with experimental methods and more recently with immunoinformatics approaches has been popular due to its usefulness for monoclonal anti-peptide Ab generation, there are reasons to focus more on discontinuous epitopes. Firstly, it is estimated that these account for 90% of all B-cell epitopes [144]. Secondly, the distinction between continuous and discontinuous epitopes is blurred, as a linear epitope with only a few Ab-binding “key” residues can be called functionally discontinuous [145]. As pointed out before, both types of antigenic determinants are actually “conformational” with respect to their functional dependence on the native protein fold.

Discontinuous epitopes are spatially clustered, surface-exposed arrangements of amino acid residues, requiring the availability of a structural model in order to be localized. The exponential rise in the number of experimentally determined and deposited macromolecular structures during the last decades [146] has made this requirement achievable. In analogy to linear epitope resources like Bcipep, databases of discontinuous epitopes have been established [147,148].

##### 4.4.2.2 Methods

One of the early methods originally targeted at linear B-cell epitopes is in fact structure-based: Thornton et al. [149] describe an algorithm to predict antigenicity by assigning a protrusion index (PI) to each residue of a protein. The PI is determined by calculation of ellipsoid parameters from the 3-dimensional protein shape, namely the orientation of the three ellipsoid axes and their length ratios. Altering the absolute length of all axes, the ellipsoid is then varied in size to contain all of the atomic positions or less. If reducing the size, the residues most protruding from the overall shape will lie outside the ellipsoid first and will get the highest PI, (e.g., 9 if they protrude from an ellipsoid that covers 90% of all atoms). The authors found that – expectedly – the PI correlates most with the surface accessibility, while its predictive power was closest to the mobility scale mentioned before.

Rapberger et al. [150] describe a computational protocol using 26 structures of Ab-antigen complexes. For each antigen structure they combined an extended accessibility criterion (with 3 Å probe radius to mimic features of the Ab) with a shape complementarity analysis to identify the best fitting paratope out of a generic structure-derived library. For the identified Ab-antigen pair, they localized the most likely contact residues, i.e., the constituents of the discontinuous epitope, by means of free binding energy estimations.

Statistical analyses of structural data can be used for the dissection of intrinsic epitope features as opposed to the non-epitope protein surfaces: Rubinstein et al. [151] evaluated a dataset containing 53 structures of Ab-antigen complexes. After dividing the derived antigen surfaces into epitope and non-epitope regions by means of an intermolecular contact criterion, they performed statistical *G*-tests on a variety of physicochemical and structural properties. Epitopes were found to be significantly enriched with aromatic and polar/charged amino acid types, and to favor certain residue pairs, frequently involving Tyrosine. Structural and geometric findings included a depletion of α-helices and β-sheets in favor of loops and flatly shaped epitope patches located on protruding parts of the molecular surface and with a high solvent accessibilities at the atomic level – observations that nicely correlate to earlier studies [149,152].

Haste-Andersen et al. [153] present an alternative dissection approach. The linear method of log-odds ratio-based propensity calculation from sequence matrices (*cf.* BepiPred above) was transferred to the structural context in several ways: firstly, the basis for training and validation was a compiled data set of 76 discontinuous epitopes and non-epitopes from X-ray structures. Secondly, epitope propensity scores were calculated as spatial C_α_ proximity sums of the raw log-odds ratios (within 10 Å radius). Thirdly, for the final prediction score the propensities were combined with distance-based intramolecular contact numbers of C_α_ atoms. Using statistical t-tests the authors had found that these contacts are significantly depleted in epitopes, which can be explained with a preference for protruding parts of the protein. Interestingly, the results of Haste-Andersen and Rubinstein exhibit considerable differences in the epitope propensity ranking of several amino acid types.

Two recent publications [154,155] describe the logistic regression and the random forest models, respectively, as classification engines. In the first work, the authors dissect epitopes by lowered temperature factors (albeit less flexibility of Ab-binding residues can be expected only in the actual Ab complex, and not in the unbound antigen, *cf.* [129]) and increased relative accessible surface area. The study of Zhang et al. [155] proposes a new criterion based on the finding that closest neighbors are more distant to the central residue in epitopes as compared to non-epitope surface patches. Thereby, an enhanced prediction performance was observed when taking the influence of epitope-adjacent internal residues into account. Finally, the authors correct for the imbalance in usual training data sets (far less epitope than non-epitope residues) by means of embedding their classification into a bootstrapping and voting procedure.

The exploitation of experimental (serological) cross-reactivity information [156], essentially indicating an identical or very similar binding mode of two antigens to the same Ab, gives rise to a fundamentally different prediction strategy, particularly suited for allergens: the comparison of physicochemical and geometrical surface features between two cross-reactive molecules directly delineates regions of high similarity as likely epitopes [157]. Dall’Antonia et al. followed this idea and developed a protocol for the comparison of 3D-aligned, feature-annotated surfaces and subsequent spatial clustering of similarity-filtered residues [158]. A key feature of this method is the weighting of similarity scores with cross-reactivity values when combining multiple pair-wise comparison results. This approach is orthogonal to most of the traditional methods in that it does not require a trained classification and is independent of propensity scores.

##### 4.4.2.3 Software implementations

Among the earliest publicly available structure-based tools, CEP [159] actually involves a hybrid linear/discontinuous prediction procedure. Linear antigenic determinants are first identified as sequentially contiguous groups of solvent accessibility-filtered residues. Conformational epitopes are then localized by means of collapsing the linear segments within a spatial range of 6 Å, while the remaining spatially isolated segments define continuous epitopes.

DiscoTope [153] is the implementation of Haste-Andersen’s prediction method, as discussed before. BePro (formerly named Pepito) [160] uses the same proximity-summed epitope propensity scales and combines them linearly with the two half-sphere exposure terms introduced by Hamelryck [161]. To increase robustness, the final residue score used for the prediction is a sum obtained from a multiple distance-threshold iteration applied to all three terms.

ElliPro [162], utilizing the previously described ellipsoid method by Thornton et al. [149], is a training-independent tool. Residues are filtered by a PI threshold and then subject to a tree-step spatial clustering algorithm. At the PI calculation stage the only change to the original algorithm is the use of residue centers-of-mass instead of C_α_ atom positions. In the absence of an input structure, the web service can be provided with the corresponding sequence for the creation of a homology model using external software.

Rubinstein et al. have developed a tool named Epitopia [163], which is unique in its dual applicability to sequence and structural input, producing linear or discontinuous epitope predictions, respectively. The developers use a Naïve Bayes classifier trained on the corresponding structure and sequence-based data sets with parameters stemming from the various features analyzed in their previous study [151]. In case of structural input, every surface-exposed residue is scored separately whereupon the immunogenicity scales are mapped to the surface for visual epitope prediction.

In the SEPPA variant of trained classification [164] pre-calculated propensity indices are derived on a spatial basis by means of comparing triangular groups of epitope and non-epitope surface residues with respect to 455 combinatorial feature patterns. In the actual prediction, all triangles within 15 Å around a probe residue are build and their average score is calculated by mapping the pre-calculated values. For the final prediction, the propensities are combined with a clustering coefficient accounting for the spatial compactness among neighbor residues around the probe.

EPSVR [165] implements a support vector regression (SVR) algorithm trained on six attributes: residue epitope propensity, conservation score, side chain energy score, contact number, surface planarity score and secondary structure composition. The method was trained with 48 pairs of Ab-bound/unbound antigen structures and tested against the CED [147]. In the prediction, the SVR model is used to determine fractions of likely epitope members in 20-residue patches. Resulting surface residue scores are the average fractional scores of all patches containing the respective probe residue.

**Fig. 6:**
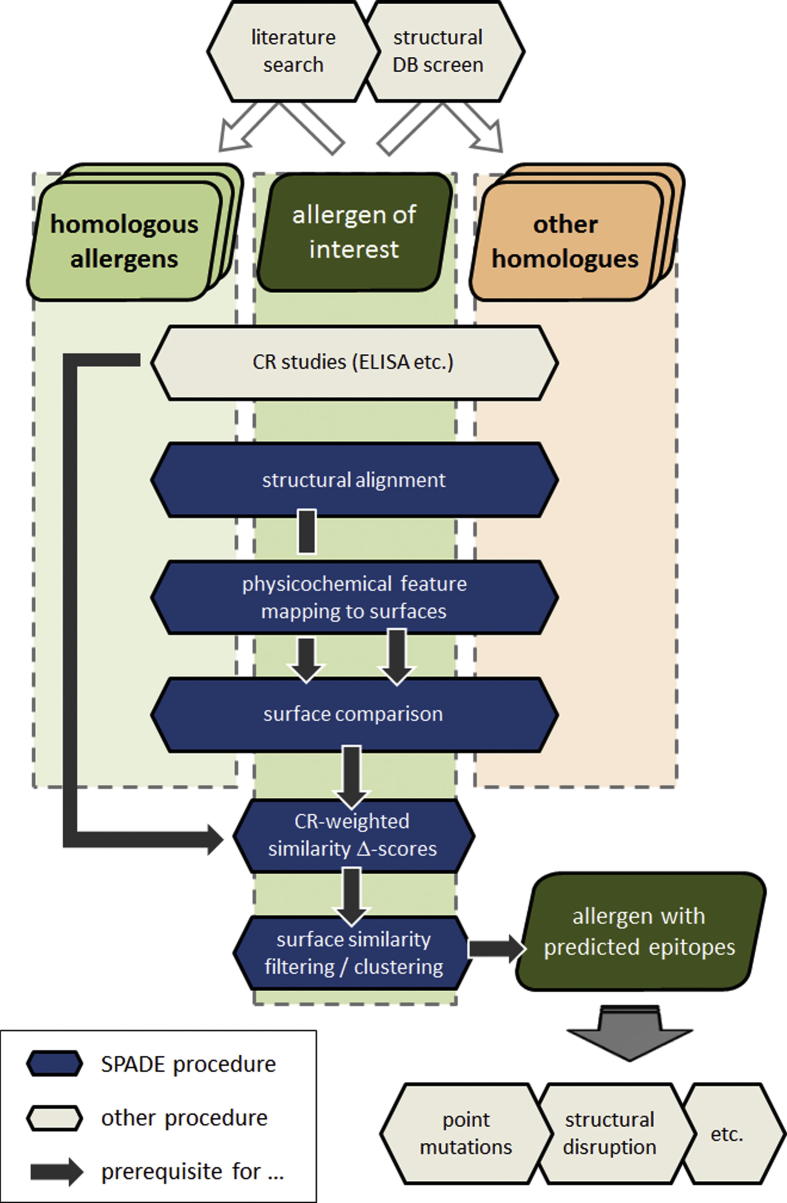
Workflow of a typical IgE epitope localization project with SPADE as prediction tool. In this procedural description starting from a target allergen with available 3D coordinates, the structure determination of homologous allergens is not considered part of the project. It is instead assumed that SPADE is applied in the presence of other pre-determined structures, which can be revealed or confirmed with literature and/or database searches. The actual computational part of the project can/should be supported by immunological data, as quantitative cross-reactivity (CR) values increase the prediction accuracy. Once epitopes and amino acid key residues for IgE-binding are located, the prediction serves as a starting point for follow-up experiments such as point mutations with tests for reduced IgE binding.

Dall’Antonia et al. implemented their approach to the SPADE tool [158], a suite of (partly 3rd party) programs for multiple pair-wise 3D-alignments and surface comparisons. The tool is provided with at least *N* = 2 allergen structures and *N* – 1 cross-reactivity values; based on this input it identifies the amino acids with highest probability for cross-reactive Ab-binding – if these are spatially contiguous they are predicted as members of a discontinuous epitope. The use of SPADE is illustrated in the flowchart of Fig. 6.

#### 4.4.3 Application of computational methods and prediction software to allergens

None of the prediction tools reviewed here, except for SPADE, have been developed with a specific application to allergens in mind. In fact it is an old question if and how allergens are intrinsically different from other antigens [166]. Structural analysis has at least provided strong evidence for the fact that allergens adopt only a restricted number of the known 3-dimensional folds and biological functions [38].

Allergen-specific resources and databases exist in which some, mostly continuous, IgE epitope information [167,168] is included. The knowledge of clinically proven allergens and the more recent availability of sufficient amounts of experimental IgE data has been utilized for sequence-based methods, such as sequence window and motif searches, to predict potentially allergenic molecules and their linear IgE epitopes [169]. A structural bioinformatics study by Jenkins et al. has reported a correlation between allergenic cross-reactivity and conserved main chain conformation as well as surface residue conservation in the presence of low overall sequence identity [170]. Furmonaviciene et al. compiled homologous allergenic and non-allergenic protein sequences for structurally determined representative allergens and used the ConSurf web service [171] to map allergen-specific conservation patterns onto the surfaces of these allergens. Thereby the authors observed characteristic patches of both highly conserved and highly variable residues in which hydrophobic amino acids were overrepresented [172].

**Fig. 7:**
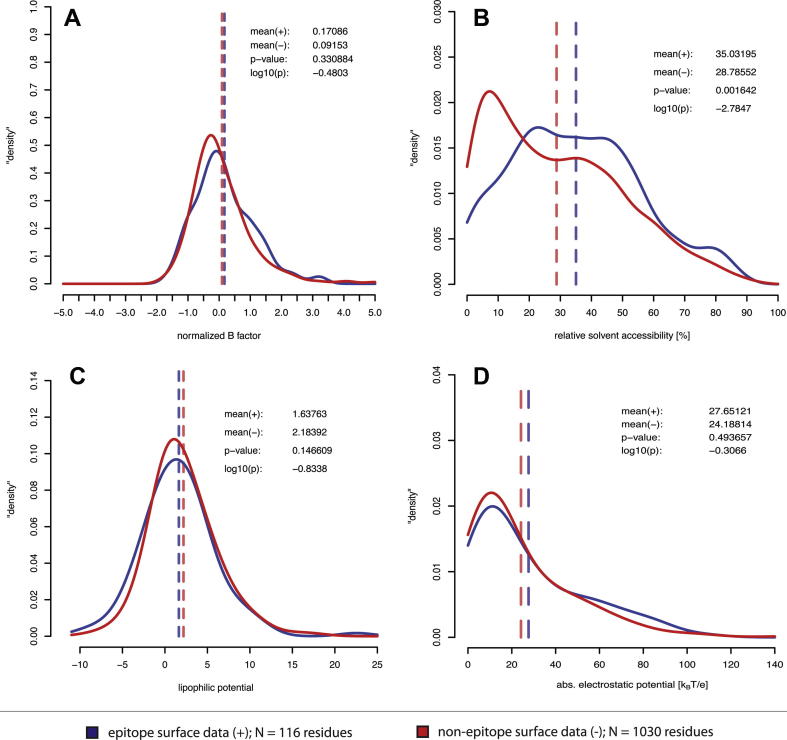
Distribution of physicochemical features in sets of epitope vs. non-epitope surface residues, represented by probability density graphs (Kernel density estimation with R), based on the seven unbound allergen structures HEL (PDB: 1VDQ), Bet v 1 (1BVQ), Hyaluronidase (1FCQ), BLG (1BEB), Bla g 2 (1YG9), Phl p 2 (1WHO) and Der f 1 (3D6S). A: Normalized B-factors of C-α atoms (all residues, separate normalization per structure) were calculated as B-norm = (B–<B>)/σ(B). B: Relative solvent accessibility S [%] was calculated as S = SASA(obs.) / SASA (max.) where SASA(obs.) is the solvent-accessible surface area of a residue X_i_ calculated with GETAREA and SASA(max.) is the corresponding area in a G-X-G tripeptide, averaged over 30 conformations [174]. C: Atomic lipophilicity parameters were taken from Ghose et al. [175], mapped to the surface according to the procedure of Heiden et al. [176] and averaged per residue (for surface vertices only). D: The electrostatic potential was calculated with APBS [177], mapped to the surface vertices and averaged per residue. Absolute quantities of [k_B_T/e] were used for the statistical analysis. A-D: Two-sided two-sample Kolmogorov–Smirnov tests were performed with R. The statistical significance of differences was defined with a confidence threshold of 0.99, so that it requires log_10_(p) ⩽ −2. Dashed lines indicate mean values.

To address the question about allergen epitope properties we revisited the epitopes defined by the structural determination of allergen-Ab complexes (Section 4.2). We performed a statistical analysis of the corresponding unbound allergen structures in order to dissect features of these allergen epitopes, as opposed to the non-allergenic surface regions. The analysis excluded the Der p 1 structure due to the fact that the 81% sequence identity to Der f 1 would corrupt the independence of data. Thus, the calculation of unique data was based on a set of 116 amino acid residues belonging to epitopes and another set of 1030 non-epitope residues located on the surfaces of seven molecules. Our study addressed the four features flexibility, solvent accessibility, lipophilicity and electrostatic potential. The statistical significance of differences in the feature distributions was assessed with Kolmogorov–Smirnov tests. The results are presented in Fig. 7, where the caption provides methodological details on how the physicochemical data were created.

Except for the significantly increased solvent accessibility in epitopes there were no significant deviations. Still, the amino acids belonging to epitopes were found to be slightly more flexible and less lipophilic than the residual surface and to exhibit a minute excess of absolute electrostatic potential. In a second analysis we used the allergen molecules directly isolated from the complex structures in order to assess the same features, but now given the binding conformations. Thereby we obtained very similar results; only the flexibility, as reflected by the temperature factors, was significantly lower than for the non-epitope surface (data not shown). The latter observation is however in agreement with the conformational fixation of the Ab-binding residues in the complexes. Our findings require two additional comments: Firstly, the amount of data is obviously not large enough to allow for statistical significance except where the feature distributions are clearly different, and the very different sample sizes in the tests pose a general statistical problem. Secondly, most of the physicochemical properties of an amino acid are attributed to the side chain, but the surprisingly high degree of exclusive backbone interaction in the Ab complexes (*cf.* section 4.2) might explain some of the observed indifference, since only the actual surface contribution of the residues was taken into account in our analysis.

The formal look at the amino acid types of Ab-binding residues, ignoring the actual contribution of atoms, leads to a similar finding as reported previously for the general antigen case [151,153], namely that all charged and some of the aromatic residues are overrepresented in epitopes. In our analysis Tryptophan, Arginine, Glutamate, Asparagine and Aspartate are the most enriched types relative to the non-epitope surfaces, with observed ratios of 3.1, 1.9, 1.6, 1.4 and 1.4, respectively.

While the lack of IgE-allergen-complex structures impedes the development of an allergen-specific prediction tool on the basis of trained classification methods, the existing software of this kind has occasionally been applied to allergens and reported in the context of either re-localization of known epitopes or – more rarely – the identification of novel antigenic determinants: Padavattan et al. applied the CEP tool to compare the predictions to the structural determinations for epitopes of bee venom hyaluronidase [120] and the grass pollen allergen Phl p 2 [81]; the latter was additionally subject to DiscoTope. CEP could in both cases locate the epitopes; for Phl p 2 one of the six epitopes predicted by CEP covered 86% of the true residues while DiscoTope could only detect 29%.

Niemi et al. applied several linear prediction tools and DiscoTope in order to re-localize the BLG epitope, which they had structurally identified in the IgE complex [116]. In their hands, all of the tools failed to predict more than one single residue correctly. This fact was explained with the nature of the discontinuous BLG IgE epitope, where most of the binding residues are located on a flat and depressed β-sheet so that the overall protrusion and/or atomic accessibilities are under average.

The SPADE authors validated their own IgE epitope predictions for BLG and Phl p 2 [158]. The BLG epitope was re-localized with 33% of the true residues and 42% specificity. For Phl p 2, 57% of the true residues were covered at a specificity of 71%. This prediction performance was compared to the other tools covered here (except BEPro and EPSVR) and found to be highest in specificity for both allergens. The best coverage (sensitivity) for Phl p 2 was obtained with SPADE as well, whereas CEP achieved the highest coverage of the BLG epitope (67%), followed by ElliPro.

In a recent study, Nair et al. [173] predicted both B- and T-cell related antigenic determinants for a presumably allergenic alcohol dehydrogenase from the mold *C. lunata* (CADH). Concerning the B-cell epitopes, a variety of linear tools were applied to the CADH sequence, while CEP, DiscoTope and ElliPro were used on a homology model. Six B-cell epitopes were predicted altogether; peptides corresponding to four of these could be experimentally confirmed as strong IgE binders. It could also be shown that the structure-based prediction tools had been able to localize three or even all four of the IgE epitopes, whereas most of the linear methods had a much weaker performance.

## 5 Conclusions

In spite of the fast growing number of allergen structures and the variety in folds observed, it has become evident that allergens belong to a limited number of fold families. Analysis of the allergen-antibody complexes provides a direct view of the experimental epitopes. The amino acid composition and the resulting physicochemical properties (flexibility, solvent accessibility, lipophilicity and electrostatic potential) are quite diverse, so that no common rules for allergen-antibody binding sites could be derived. This is not surprising with respect to the low number of non-redundant complex structures available. In conclusion, more experimental data (i.e. allergen-IgE complexes) are necessary for a direct visualization of binding sites as well as for the verification of prediction methods. On the other hand the recent developments of structure based prediction tools provided promising results towards higher accuracy and reliability.

## Figures and Tables

**Table 1:** Structures of allergens deposited in the PDB determined by X-ray crystallography or NMR grouped by Pfam families.

Allergen	Species common name	Species scientific name	X-ray structure	NMR	References
*Bet_v_1 (PF00407): Pathogenesis-related (PR10) protein/Bet v 1 family*					
Api g 1	Celery	*Apium graveolens*	2BK0		[178]
Bet v 1	White birch	*Betula verrucosa*	1LLT, 1BV1, 1FM4, 1QMR, 1FSK, 4A80–8, 4A8G, 4A8U, 4A8 V	1BTV, 1B6F	[41,179–184]
Dau c 1	Carrot	*Daucus carota*	2WQL		[185]
Fra a 1	Strawberry	*Fragaria x ananassa*		2LPX	-
Gly m 4	Soybean	*Glycine max*		2K7H	[186]
Pru av 1	Sweet cherry	*Prunus avium*		1E09, 1H2O	[126]
Vig r 6.0101	Mung bean	*Vigna radiata*	2FLH, 3C0 V		[187]
LLR18A/ LLR18B	Yellow lupine	*Lupinus luteus*	1ICX, 1IFV		[188]
LIPR-10.2.B	Yellow lupine	*Lupinus luteus*	2QIM		[189]

*Tryp_alpha_amyl (PF00234): Protease inhibitor/seed storage/lipid transfer proteins (LTP) family*					
Ara h 2	Peanut	*Arachis hypogaea*	3OB4		[10]
Ara h 6	Peanut	*Arachis hypogaea*		1W2Q	[30]
Ber e 1	Brazilian nut	*Bertholletia excelsa*		2LVF	[190]
Bra n 1	Rapeseed	*Brassica napus*		1PNB	[191]
Hor v 1	Barley	*Hordeum vulgare*		1JTB, 1BE2	[192,193]
Pru p 3	Peach	*Prunus persica*	2ALG, 2B5S		[46]
Ric c 3	Castor bean	*Ricinus communis*		1PSY	[43]
Zea m 14	Corn	*Zea mays*	1MZL, 1MZM, 1FK0–7	1AFH	[194–196]

*Cupin_1 (PF00190): Cupin superfamily*					
Ara h 1	Peanut	*Arachis hypogaea*	3S7E, 3S7I, 3SMH		[197–199]
Ara h 3	Peanut	*Arachis hypogaea*	3C3 V		[200,201]
Gly m 6.0101	Soybean	*Glycine max*	1FXZ		[202]
Gly m 6.0501	Soybean	*Glycine max*	1OD5, 2D5F, 2D5H		[203,204]
Gly m conglycinin	Soybean	*Glycine max*	1IPK, 1IPJ		[205]
Pru du 6	Almond	*Prunus dulcis*	3FZ3		[206,207]

*Lipocalin (PF00061): Lipocalin/cytosolic fatty-acid binding protein family*					
Arg R 1	Pigeon tick	*Argas reflexus*	2X46, 2X45		-
Bla g 4	German cockroach	*Blattella germanica*	3EBK		[55]
Bos d 2	Cow	*Bos taurus*	1BJ7		[208]
Bos d 5	Cow	*Bos taurus*	1BSO		[209]
Can f 2	Dog	*Canis familiaris*	3L4R		[54]
Der f 13	House dust mite	*Dermatophagoides farinae*		2A0A	[210]
Equ c 1	Horse	*Equus caballus*	1EW3		[211,212]
Mus m 1	Mouse	*Mus musculus*	1MUP, 1JV4	1DF3	[213–215]
Per a 4	American cockroach	*Periplaneta americana*	3EBW		[55]
Rat n 1	Rat (urine)	*Rattus norvegicus*	2A2U, 2A2G		[216]

*EF hand (PF00036): EF hand family*					
Bet v 4	White birch	*Betula verrucosa*		1H4B	[31]
Che a 3	Lamb’s Quarters, White goosefoot	*Chenopodium album*	2OPO		[62]
Cyp c 1	Carp	*Cyprinus carpio*	4CPV, 5CPV and other∗		[58,59]
Phl p 7	Timothy-grass	*Phleum pratense*	1K9U	2LVK, 2LVJ, 2LVI	{Verdino, 2002 #13[63]

*Peptidase_C1 (PF00112): Papain-like cysteine protease*					
Act d 1	Kiwi fruit	*Actinidia delicionsa*	2ACT, 1AEC		[217–219]
Car p 1	Papaya	*Carica papaya*	1PPN, 1KHQ		[220,221]
Der p 1	House dust mite	*Dermatophagoides pteronyssinus*	1XKG, 2AS8, 3F5 V, 3RVX, 3RVW		[66,115,222]
Der f 1	House dust mite	*Dermatophagoides farinae*	3D6S, 3RVV		[115,223]

*Profilin (PF00235): Profilin family*					
Ara h 5	Peanut	*Arachis hypogaea*	4ESP		[224]
Ara t 8	Mouse-ear crest	*Arabidopsis thaliana*	1A0 K		[225]
Bet v 2	White birch	*Betula verrucosa*	1CQA		[226]
Hev b 8	Latex	*Hevea brasiliensis*	1G5U		-
Thaumatin (PF00314): Thaumatin-like protein					
Act d 2	Kiwi fruit	*Actinidia deliciosa*	4BCT		-
Mal d 2	Apple	*Malus x domestica*	3ZS3		-
Mus a 4	Banana	*Musa acuminata*	1Z3Q		[74]
Pru av 2	Sweet cherry	*Prunus avium*	2AHN		[73]

*Pollen_allerg_1 (PF01357): Expansin, C-terminal domain*					
Phl p 1 (C-term domain)	Timothy-grass	*Phleum pratense*	1N10		[78]
Phl p 2	Timothy-grass	*Phleum pratense*	1WHO, 1WHP, 2VXQ	1BMW	[15,81]
Phl p 3	Timothy-grass	*Phleum pratense*	3FT1, 3FT9	2JNZ	[80]
Zea m 1 (C-term domain)	Corn	*Zea mays*	2HCZ		[79]

*DPBB_1 (PF03330): rare lipoprotein A (RlpA)-like double-psi beta-barrel; Expansin, N-terminal domain*					
Phl p 1 (N-term domain)	Timothy-grass	*Phleum pratense*	1N10		[78]
Zea m 1 (N-termn domain)	Corn	*Zea mays*	2HCZ		[79]

*Pollen_allerg_2 (PF01620): Ribonuclease; Group 5/6 grass pollen allergen*					
Phl p 5b	Timothy-grass	*Phleum pratense*	1L3P		[83]
Phl p 6	Timothy-grass	*Phleum pratense*	1NLX		-

*Blo-t-5 (PF11642): Group 5/21 mite allergen*					
Blo t 5	Storage mite	*Blomia tropicalis*		2JMH, 2JRK	[27,29]
Der p 5	House dust mite	*Dermatophagoides pteronyssinus*	3MQ1		[84,227]
Blo t 21	Storage mite	*Blomia tropicalis*		2LM9	[228]

*CAP (PF00188): CRISP; PR-1; antigen 5 (Ag5)*					
Sol i 3	Fire ant	*Solenopsis invicta*	2VZN		[229]
Ves v 5	Common wasp	*Vespula vulgaris*	1QNX		[87]

*Chitin_bind_1 (PF00187): Chitin recognition protein; Hevein-like domain*					
Hev b 6.02	Latex	*Hevea brasiliensis*	1WKX, 1Q9B	1HEV, 1T0 W	[89,230]
Tri a 18	Wheat	*Triticum aestivum*	1WGC, 2CWG, 7WGA, 9WGA, 2UVO, 4AML, 2X3T		[231–233]

*E1_DerP2_DerF2 (PF02221): MD-2-related lipid-recognition (ML) domain; Group 2 mite allergen*					
Der f 2	House dust mite	*Dermatophagoides farinae*	1XWV, 2F08	1AHK, 1AHM, 1WRF	[93,94,234,235]
Der p 2	House dust mite	*Dermatophagoides pteronyssinus*	1KTJ	1A9 V	[90,92]

*Glyco_hydro_17 (PF00332): Glycosyl hydrolases family 17/endo-1,3-beta-glucosidase*					
Hev b 2	Latex	*Hevea brasiliensis*	3EM5, 3F55		-
Mus a 5	Banana	*Musa acuminata*	2CYG		[96]

*Glyco_hydro_56 (PF01630): Hyaluronidase*					
Api m 2	Honey bee	*Apis mellifera*	1FCQ, 1FCU, 1FCV, 2J88		[98,120]
Ves v 2	Common wasp	*Vespula vulgaris*	2ATM		[99]

*Pro_isomerase (PF00160): Cyclophilin type peptidyl-prolyl cis–trans isomerase/CLD*					
Asp f 11	Mold	*Aspergillus fumigatus*	2C3B		[105]
Mala s 6	Mold	*Malassezia sympodialis*	2CFE		[104]

*FAD linked oxidase (PF01565) / BBE-like (PF08031)*					
Cyn d 4	Bermuda grass	*Cynodon dactylon*	4DNS		[106]
Phl p 4	Timothy-grass	*Phleum pratense*	3TSH, 3TSJ		[107]

Many parvalbumin structures have been solved (37 PDB entries currently) comprising proteins from 9 different species (from fish to human). Allergenicity has been determined for most fish parvalbumins and the structures of the carp parvalbumins are listed as representatives for this group.

**Table 2:** Structures of allergens deposited in the PDB determined by X-ray crystallography or NMR with only one structure from the Pfam family determined (or Pfam family not known).

Allergen	Function/fold	Species common name	Species scientific name	X-ray structure	NMR	References
Aed a 2		Yellow fewer mosquito	*Aedes aegypti*	3DXL, 3DY9, 3DYE, 3DZT		[236]
Alt a 1		Mold	*Alternaria alternata*	3V0R		[237]
Amb t 5		Giant ragweed	*Ambrosia trifida*		1BBG, 2BBG, 3BBG	-
Api m 1	Phospholipase A2	Honey bee	*Apis mellifera*	1POC		[238]
Api m 4	Melittin	Honey bee	*Apis mellifera*	2MLT	1BH1	[239–241]
Art v 1	Gamma-thionin (plant defensin)	Mugwort	*Artemisia vulgaris*		2KPY	[242]
Asc s 1	ABA-1 polyprotein	Roundworm of pigs	*Ascaris suum*		2XV9	[125]
Asp f 1	Ribonuclease	Mold	*Aspergillus fumigatus*	1AQZ		[243]
Asp f 6	Fe, Mn superoxide dismutase	Mold	*Aspergillus fumigatus*	1KKC		[244]
Asp o 21	TAKA-amylase A	Mold	*Aspergillus oryzae*	7TAA		[245]
Bla g 2	Aspartic protease	German cockroach	*Blattella germanica*	2NR6, 1YG9, 3LIZ		[119,246,247]
Bos d 4	Alpha-lactalbumin	Cattle	*Bos taurus*	2G4 N, 1F6R, 1F6S, 1HFZ		[248,249]
Chi t 1	Globin	Midge	*Chironomus thummi thummi*	1ECO		[250]
Cla h 8	Mannitol dehydrogenase	Mold	*Cladosporium herbarum*	3GDF, 3GDG		[251]
Der p 7	Extracellular solute-binding protein	European house dust mite	*Dermatophagoides pteronyssinus*	3H4Z		[252]
Der f 7		House dust mite	*Dermatophagoides farinae*	3UV1		[9]
Equ c 3	Serum-albumin	Horse	*Equus caballus*	3V08		-
Fel d 1	Uteroglobin	Cat (saliva)	*Felis catus*	2EJN, 1PUO, 1ZKR		[253,254]
Gal d 3	Transferrin	Chicken	*Gallus domesticus*	1NNT, 1OVT, 1AIV, 1RYX, 1N04, 1IEJ, 2D3I		
Gal d 4	Lysozyme	Chicken	*Gallus domesticus*	>50 entries		
Gly m lectin	Legume lectin	Soybean	*Glycine max*	1SBF		[255]
Jun a 1	Pectate lyase	Mountain cedar	*Juniperus ashei*	1PXZ		[256]
Mala s 1	Maltose-binding protein	Mold	*Malassezia sympodialis*	2P9 W		[257]
Mala s 13	Thioredoxin	Mold	*Malassezia sympodialis*	2J23		[258]
Ole e 6		Olive	*Olea europaea*		1SS3	[28]
Ole e 9	Beta-1,3-glucanase	Olive	*Olea europaea*		2JON	[259]
Ovalbumin	Serine-protease inhibitor (serpin)	Chicken	*Gallus gallus*	1UHG, 1JTI, 1OVA		[260,261]
Per a 4	Lipocalin-like	American cockroach	*Periplaneta americana*	3EBW		[55]
Sol i 2		Fire ant	*Solenopsis invicta*	2YGU		[262]

**Table 3:** Structures of complexes between allergens and monoclonal antibodies with characteristics of interaction.

Allergen	mAb F_ab_^a^	PDB-id	*d*^b^	Buried interface area^c^	CDR^d^ involved	Number of ...					
						Functional epit. res.	Structural epit. res.	Linear segments^e^	Hydrogen bonds	Ionic interactions^f^	Apolar contacts
HEL	HyHEL8 (IgG)	1NDG	1.9 Å	1827 Å^2^	L1, L2, L3H1, H2, H3	8	21	4	13	2	38
Bet v 1	BV16 (IgG)	1FSK	2.9 Å	1649 Å^2^	L1, L3H1, H2, H3	7	17	1	11	3	34
Api m 2 (hyaluronidase)	21E11 (IgG)	2J88	2.6 Å	1529 Å^2^	L1, L2, L3H1, H2, H3	8	10	1	13	2	27
BLG	D1 (IgE F_v_)	2R56	2.8 Å	1750 Å^2^	L1, L2, L3H1, H2, H3	9	21	5	13	3	28
Bla g 2	7C11 (IgG)	2NR6	2.8 Å	1773 Å^2^	L1, L2, L3H1, H2, H3	12	16	3	20	4	23
Phl p 2	HuMab2 (IgE F_v_)	2VXQ	1.9 Å	1616 Å^2^	L1, L3H1, H2, H3	9	21	4	14	1	26
Der f 1	4C1 (IgG)	3RVV	1.9 Å	1385 Å^2^	L1, L2, L3H1, H2, H3	5	15	2	7	2	26
Der p 1	4C1 (IgG)	3RVW	2.0 Å	1371 Å^2^	L1, L2, L3H1, H2, H3	6	15	2	11	2	21

a mAb: name of the monoclonal antibody and its immunoglobulin type. Fab = antigen-binding fragment. Fv: variable part of the antibody respectively Fab.

b *d*: crystallographic resolution of the structure.

c Δ(SASA) = [SASA(Ab) + SASA(allergen)] – SASA(complex); SASA = solvent accessible surface area as calculated with GETAREA [263] using a 1.4 Å probe radius.

d CDR: complementarity determinant region; counted as involved if forming at least one specific interaction. L = light chain loops, H = heavy chain loops.

e Linear segments constituting the epitopes are defined here as continuous sequence stretches of at least three residues (with single position gaps allowed).

f Ionic interactions include both short-distance contacts with H-bond contribution (salt bridges) and weaker electrostatic-only interactions. Both ionic and apolar interactions were determined at a distance cutoff of 4.0 Å and counted once per allergen–Ab residue pair. Hydrogen bonds were determined at a cutoff of 3.25 Å.
